# Manufacturing cost analysis and exploratory economic evaluation of natural killer cell immunotherapy for paediatric solid tumors

**DOI:** 10.1002/cti2.70122

**Published:** 2026-09-20

**Authors:** Sabera Tabassum Wahid, Melissa Elliott, Yufan Wang, Wayne Nicholls, Natacha Omer, Fernando Souza‐Fonseca‐Guimaraes, Haitham Tuffaha

**Affiliations:** ^1^ Centre for the Business and Economics of Health The University of Queensland Brisbane QLD Australia; ^2^ Frazer Institute, Faculty of Health, Medicine and Behavioural Sciences The University of Queensland Woolloongabba QLD Australia; ^3^ Oncology Services Group Queensland Children's Hospital South Brisbane QLD Australia; ^4^ School of Pharmacy and Pharmaceutical Sciences The University of Queensland Woolloongabba QLD Australia; ^5^ Cancer Council Queensland Brisbane QLD Australia

**Keywords:** cell therapy manufacturing, cost‐effectiveness analysis, early economic evaluation, micro‐costing, natural killer cell therapy, paediatric solid tumors

## Abstract

**Objectives:**

Natural killer (NK) cell therapy is a promising allogeneic, off‐the‐shelf immunotherapy for solid tumors, yet economic evidence to guide its clinical translation is absent. This study aimed to conduct a micro‐costing analysis of their manufacturing and perform an exploratory economic evaluation of NK cell therapy for paediatric solid tumors from an Australian health system perspective.

**Methods:**

A micro‐costing analysis was conducted using primary laboratory production data across six manufacturing steps and categories. Hospitalisation costs were estimated by expert opinion. Effectiveness estimates were drawn from chimeric antigen receptor (CAR)‐T cell therapy economic evaluations. Analyses included incremental cost‐effectiveness ratios (ICERs), headroom analysis, net monetary benefit (NMB) and probabilistic sensitivity analysis.

**Results:**

Manufacturing cost was AUD 58 681 (USD 38 143) per batch (AUD 9780; USD 6357 per dose). Eighty‐eight per cent of costs were concentrated in three steps: NK cell expansion (50.8%), mRNA transfection (25.1%) and T‐cell depletion (11.7%), with consumables/reagents comprising 85% of the total production cost. Combining manufacturing (AUD 58 681) and hospitalisation (AUD 75 000; USD 48 750) costs, the total NK cell therapy cost was AUD 133 681 (USD 86 893) per patient, yielding an illustrative ICER of AUD 35 167 (USD 22 859) per QALY gained, based on a proxy effectiveness estimate. Economic headroom was AUD 56 386 (USD 36 651) per patient. Probabilistic analysis showed 51.7% probability of cost‐effectiveness at AUD 50 000/QALY, with a minimum of 2.67 QALYs required.

**Conclusion:**

NK cell therapy shows indicative early economic signals warranting further clinical and economic investigation. Micro‐costing identifies actionable manufacturing cost drivers to inform process optimisation, and these exploratory estimates provide a foundation for iterative economic evaluation as clinical evidence develops.

## Graphical abstract

Exploratory economic evaluation of allogeneic NK‐cell therapy for paediatric solid tumors, highlighting treatment costs and the clinical benefit required for cost‐effectiveness. Figure made in ©BioRender—biorender.com.

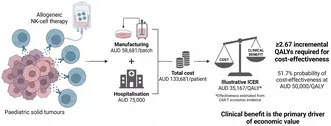

## Introduction

Paediatric solid tumors with relapsed or refractory disease, including sarcoma and neuroblastoma, represent a significant unmet clinical need, with 5‐year survival rates below 20% and limited effective treatment options.^1^, ^2^ Natural killer (NK) cells are innate cytotoxic lymphocytes that play a central role in tumor immune surveillance. Unlike T cells, NK cells recognise and eliminate target cells through detection of stress‐induced ligands or downregulated major histocompatibility complex (MHC) class I molecules, without requiring prior sensitisation or antigen specificity.^3^ This mechanism of action confers several translational advantages over Chimeric Antigen Receptor (CAR) T‐cell therapy, which, despite demonstrating remarkable efficacy in certain haematologic malignancies,^4^, ^5^ has shown limited activity in solid tumors and carries risks of serious adverse effects including cytokine release syndrome and neurotoxicity.^6^, ^7^ In contrast, NK cells can be administered allogeneic with a substantially lower risk of graft‐versus‐host disease (GvHD), as they do not require patient‐specific genetic engineering.^3^ This enables off‐the‐shelf manufacturing, in contrast to CAR‐T cell therapies, which must be produced autologously from each patient's own cells, incurring considerably higher production costs.^8^ Beyond these manufacturing advantages, NK cells recognise and eliminate malignant cells through multiple germline‐encoded activating receptors, conferring a poly‐antigen reactivity intrinsic to both allogeneic and autologous NK cells, which reduces reliance on single‐antigen targeting and the risk of antigen escape associated with CAR‐T cell therapies.^9^ In the allogeneic setting used here, mismatch between donor and recipient can further elicit an alloreactive graft‐versus‐tumor effect, analogous to the GvHD effect described in haematopoietic stem cell transplantation.^10^ Additionally, NK cells have demonstrated the ability to infiltrate metastatic lesions and contribute to anti‐tumor immune responses in solid tumor settings, suggesting potential clinical benefit in these contexts.^11^, ^12^ Importantly, this allogeneic compatibility enables off‐the‐shelf manufacturing, with the potential to improve accessibility and reduce per‐patient costs.

NK cell therapy production is a complex, multi‐step process.^13^, ^14^ Briefly, peripheral blood mononuclear cells (PBMCs) are isolated from donor peripheral blood, leukapheresis products or cord blood units (CBU).^14^, ^15^ Following this, the NK cell population is routinely enriched by depleting CD3^+^ T cells, which also minimise potential T‐cell contaminants and the risk of GvHD or CRS.^16^ Where enhanced functionality is required, genetic modification is performed via lentiviral transduction or mRNA transfection.^17^, ^18^ Finally, NK cells are expanded using feeder cells or cytokine cocktails until clinically relevant numbers are achieved, with stringent quality control conducted throughout to ensure product safety and efficacy. This manufacturing complexity results in high production costs for cell therapies. For example, CAR‐T therapy in Australia is priced between AUD 500 000 and AUD 750 000 (~USD 325 000–487 500) per patient, excluding supportive care and hospitalisation costs,^19^, ^20^ with total treatment costs exceeding AUD 1.5 million (~USD 1 million) when all associated healthcare resources are considered.^21^ While NK cell therapy is anticipated to be less costly given its allogeneic, off‐the‐shelf manufacturing model, these figures underscore the importance of rigorous cost estimation and early economic evaluation before committing to large‐scale clinical development.

Many therapies that show early clinical promise subsequently fail to demonstrate sufficient value for money, not necessarily because of lack of efficacy, but because economic barriers are identified too late in the development pathway to allow course correction. Embedding economic thinking early in technology development allows cost drivers to be identified and addressed before they become prohibitive.^22^, ^23^, ^24^ For NK cell therapies, currently in early clinical development, such evaluation serves two critical translational functions: (1) identifying manufacturing cost drivers to inform process optimisation and scale‐up; and (2) producing indicative cost‐effectiveness estimates to signal economic feasibility and define the parameters for future modelled evaluation. Despite the growing application of economic evaluation to CAR‐T and other cell therapies,^25^ no such analysis exists for NK cell therapy, leaving developers, funders and policymakers without the economic evidence needed to guide translational decisions. This study therefore aimed to (1) conduct a micro‐costing analysis of NK cell manufacturing to identify key production cost drivers; and (2) perform an exploratory economic evaluation to generate preliminary estimates of cost‐effectiveness, headroom and net monetary benefit as a foundation for future modelled evaluation.

## Results

### NK cell manufacturing cost

A detailed micro‐costing analysis identified the key cost drivers in the NK cell manufacturing process (Table 1). The total production cost was AUD 58 681 (USD 38 143) per batch, equivalent to AUD 9780 (USD 6357) per dose. At the process level, costs were heavily concentrated in three steps: NK cell expansion was the single largest contributor at 50.8% of total production costs (AUD 29 812; USD 19 378), followed by mRNA transfection at 25.1% (AUD 14 707; USD 9559) and CD3^+^ T‐cell depletion at 11.7% (AUD 6864; USD 4461), together accounting for 88% of total production costs. The remaining steps contributed 12%: feeder cell expansion (4.6%; AUD 2680), quality control analysis (3.8%; AUD 2205), cleanroom and storage (2.6%; AUD 1502) and PBMC isolation (1.6%; AUD 912). At the resource level, equipment and consumables (45.7%; AUD 26 796) and reagents (39.7%; AUD 23 317) together accounted for 85% of the cost burden, while personnel (8.2%; AUD 4811), facility (5.1%; AUD 3014) and instruments (1.3%; AUD 742) made minor contributions.

**Table 1:** Manufacturing cost analysis of NK cell therapy by process step and resource category

Component	AUD	USD^a^	Percentage of total cost
By process step			
NK cell expansion	29 812	19 378	50.8
mRNA transfection	14 707	9559	25.1
CD3^+^ T‐cell depletion	6864	4461	11.7
Feeder cell expansion	2680	1742	4.6
QC analysis	2205	1433	3.8
PBMC isolation	912	593	1.6
Cleanroom and storage	1502	976	2.6
Total production cost per batch	58 681	38 143	100
Total production cost per dose	9780	6357	—
By resource category			
Equipment/Consumables	26 796	17 417	45.7
Reagents	23 317	15 156	39.7
Personnel	4811	3127	8.2
Facility	3014	1959	5.1
Instruments	742	482	1.3
Total production cost per batch	58 681	38 143	100

Component costs are rounded to the nearest Australian dollar for presentation. Totals are calculated from unrounded source values and then rounded, and may therefore differ by up to one dollar from the sum of the displayed components.

Abbreviations: AUD, Australian dollars; PBMC, peripheral blood mononuclear cells; QC, quality control; USD, United States dollars.

^a^ Exchange rate: AUD 1 = USD 0.65.

### Exploratory economic evaluation

The base‐case exploratory economic analysis estimated a total cost of NK cell therapy of AUD 133 681 (USD 86 893) per patient, comprising AUD 58 681 (USD 38 143) in production costs and AUD 75 000 (USD 48 750) in hospitalisation costs (Table 2). Using a proxy effectiveness estimate of 3.80 incremental QALYs, the ICER was AUD 35 167 (USD 22 859) per QALY gained, below the Australian willingness‐to‐pay threshold of AUD 50 000 (USD 32 500) per QALY. Headroom analysis indicated a maximum justifiable cost of AUD 190 067 (USD 123 543) per patient, yielding economic headroom of AUD 56 386 (USD 36 651) above the estimated cost. At an annual production volume of 50 patients, this translated into population NMB of AUD 2 819 300 (USD 1 832 545).

**Table 2:** Exploratory economic evaluation results for NK cell therapy in paediatric relapsed/refractory sarcoma and neuroblastoma

Parameter	AUD	USD^a^
Production costs		
NK cell production cost per batch	58 681	38 143
NK cell production cost per dose	9780	6357
Treatment costs		
Hospitalisation costs per patient	75 000	48 750
Total cost per patient	133 681	86 893
Effectiveness		
Incremental QALYs (proxy estimate)	3.801	—
Cost‐effectiveness		
Incremental cost‐effectiveness ratio (ICER)	35 167/QALY	22 859/QALY
Willingness‐to‐pay (WTP) threshold/QALY	50 000	32 500
Headroom analysis		
Maximum allowable cost per patient	190 067	123 543
Economic headroom per patient	56 386	36 651
Population‐level net monetary benefit (NMB)		
Anticipated annual patient volume (V)	50	—
Total population‐level NMB (*V* = 50 patients/year)	2 819 300	1 832 545

Values are rounded for presentation. Derived values (maximum allowable cost and economic headroom) are calculated from the unrounded mean estimate of 3.801340 QALYs and may not reproduce exactly from the rounded figures shown.

Abbreviations: AUD, Australian dollars; ICER, incremental cost‐effectiveness ratio; NMB, net monetary benefit; QALY, quality‐adjusted life‐year; WTP, willingness‐to‐pay threshold.

^a^ Exchange rate: AUD 1 = USD 0.65.

### Uncertainty analysis

#### Manufacturing cost sensitivity analysis

**Figure 1:**
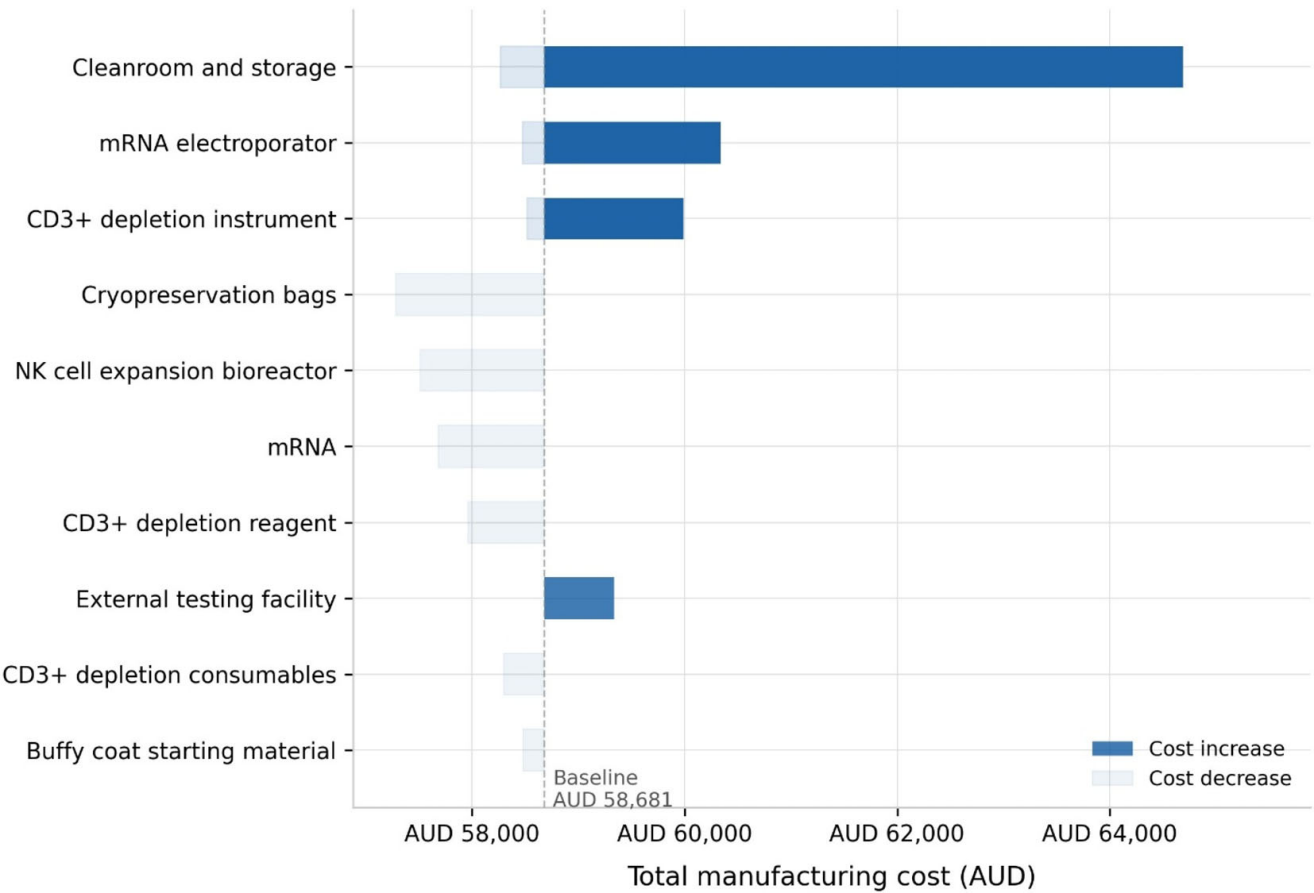
One‐way sensitivity analysis of NK cell manufacturing costs. Tornado plot showing the impact of varying key cost inputs on total manufacturing cost per batch (baseline: AUD 58 681). Variation is driven primarily by cleanroom and storage and specialised equipment used in mRNA transfection and CD3^+^ T‐cell depletion.

One‐way sensitivity analysis showed that total production cost was most sensitive to capital‐intensive inputs, particularly cleanroom and storage and specialised equipment used in mRNA transfection and CD3^+^ T‐cell depletion (Figure 1). Although the cost burden is concentrated in key manufacturing steps, cost variability is driven primarily by infrastructure and equipment, while consumables, reagents and personnel had smaller effects. This highlights the importance of capital utilisation and production scale in determining per‐batch costs.

#### Production volume threshold analysis

**Figure 2:**
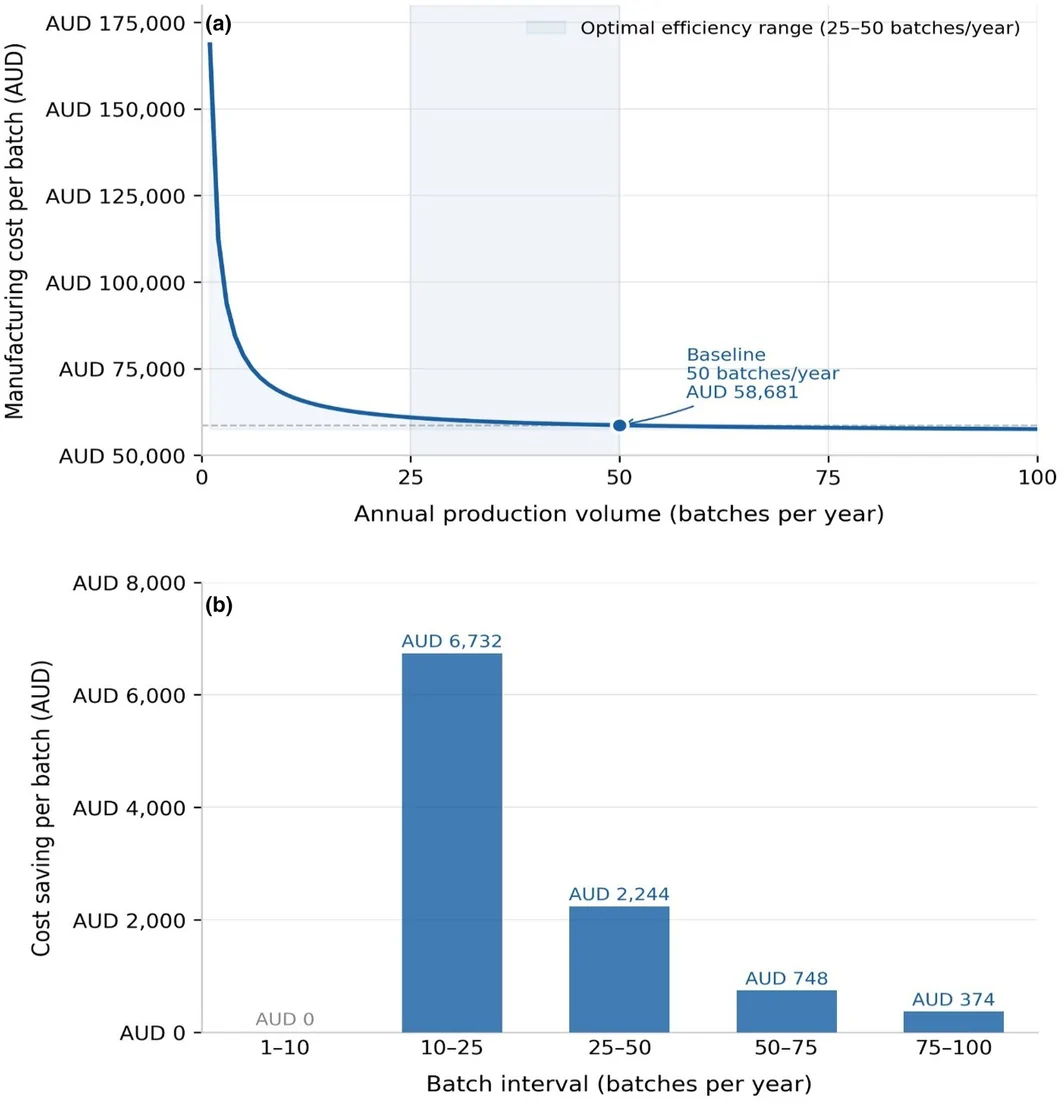
Production volume threshold analysis. **(a)** Relationship between annual production volume and per‐batch manufacturing cost, demonstrating economies of scale and identifying the optimal efficiency range. **(b)** Incremental cost savings per batch across production volume intervals, showing diminishing marginal gains at higher production volumes.

Threshold analysis revealed an inverse relationship between annual production volume and per‐batch costs, with sharp cost reductions observed between 1 and 25 batches per year and diminishing marginal savings beyond 25 batches (Figure 2a). The point of greatest cost efficiency lies at 25–50 batches per year, beyond which additional scale‐up yields minimal further savings. Incremental cost savings were greatest when scaling from 10 to 25 batches per year (AUD 6732 per batch), with smaller gains between 25 and 50 batches (AUD 2244), and diminishing economic benefits beyond 50 batches (AUD 748 between 50–75 and AUD 374 between 75–100) (Figure 2b). A separate consolidation scenario indicated that producing two patients' doses within a single manufacturing run generated savings of AUD 9649 (USD 6272) per production cycle and AUD 3216 (USD 2090) per batch, reflecting shared resources and reduced duplication of processes. Introduction of a 5% batch failure rate increased costs under both production models, although the impact was smaller under the consolidated approach, suggesting improved efficiency and resilience.

#### Economic evaluation sensitivity analysis

One‐way sensitivity analysis demonstrated that the ICER was highly sensitive to QALY gains but relatively robust to variations in hospitalisation and manufacturing costs. ICER ranged from AUD 14 176 (USD 9214) per QALY at maximum QALY gains (9.43) to AUD 145 305 (USD 94 448) per QALY at minimum gains (0.92), the latter well exceeding the AUD 50 000 threshold. Varying hospitalisation costs across the full plausible range (AUD 50 000–100 000) yielded ICERs of AUD 28 590–41 743 (USD 18 584) per QALY, remaining within acceptable thresholds throughout. Variations in manufacturing costs had minimal impact on the ICER. Threshold analysis identified a minimum QALY gain of 2.67 required for cost‐effectiveness at the AUD 50 000/QALY threshold, a maximum hospitalisation cost of AUD 131 386 (USD 85 401) per patient and a break‐even WTP of AUD 35 167 (USD 22 859) per QALY, below the AUD 50 000 Australian benchmark.

#### Headroom and net monetary benefit sensitivity analysis

**Figure 3:**
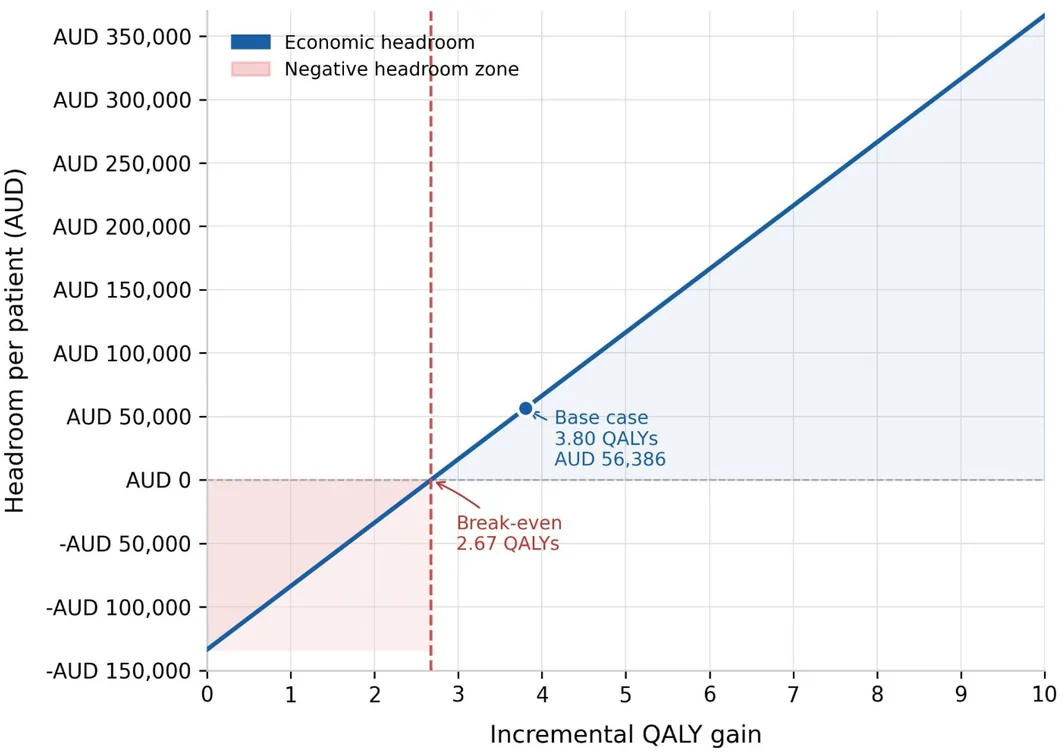
Headroom sensitivity analysis. Economic headroom per patient as a function of incremental QALY gain at a willingness‐to‐pay threshold of AUD 50 000/QALY, indicating the break‐even point and negative headroom region.

**Figure 4:**
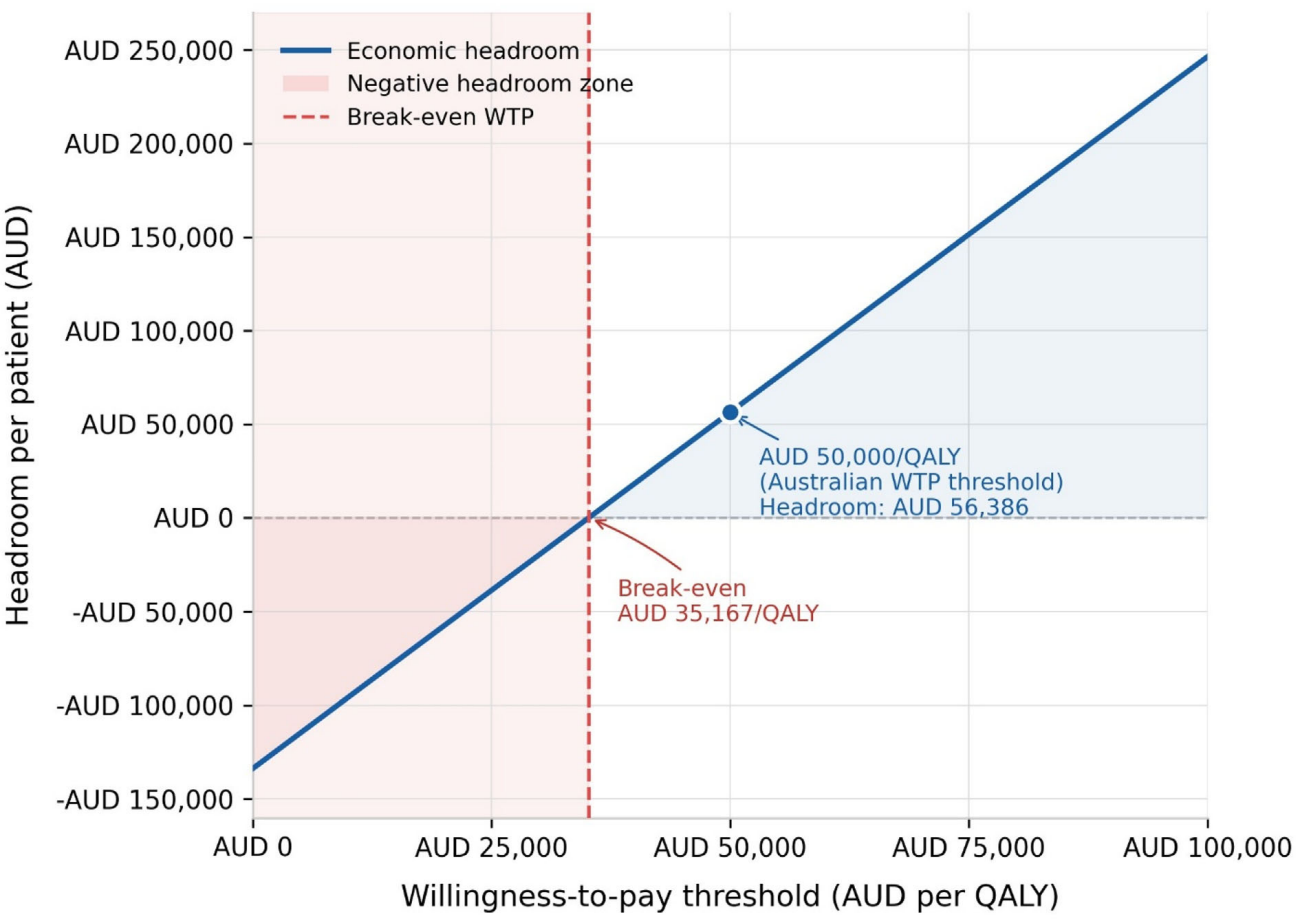
Headroom analysis across willingness‐to‐pay thresholds. Economic headroom per patient as a function of willingness‐to‐pay thresholds, indicating the break‐even threshold and negative headroom region.

Economic headroom declined markedly as QALY gains decreased, becoming negative below the 2.67 QALY threshold (Figure 3) and increased proportionally with the WTP threshold, crossing zero at AUD 35 167/QALY (Figure 4). These findings confirm that value depends primarily on realising projected clinical outcomes rather than on manufacturing cost reductions alone. At the extremes of the QALY range, net monetary benefit per patient ranged from AUD 337 819 (USD 219 582) at QALY gains of 9.43 to −AUD 87 681 (USD −56 993) at QALY gains of 0.92. Two‐way sensitivity analysis indicated that maintaining a NMB at WTP thresholds below AUD 50 000/QALY required minimum QALY gains of 3.6–3.7, somewhat higher than the cost‐effectiveness threshold of 2.67 QALYs, suggesting the investment case is more demanding than the cost‐effectiveness case alone.

#### Probabilistic sensitivity analysis

**Figure 5:**
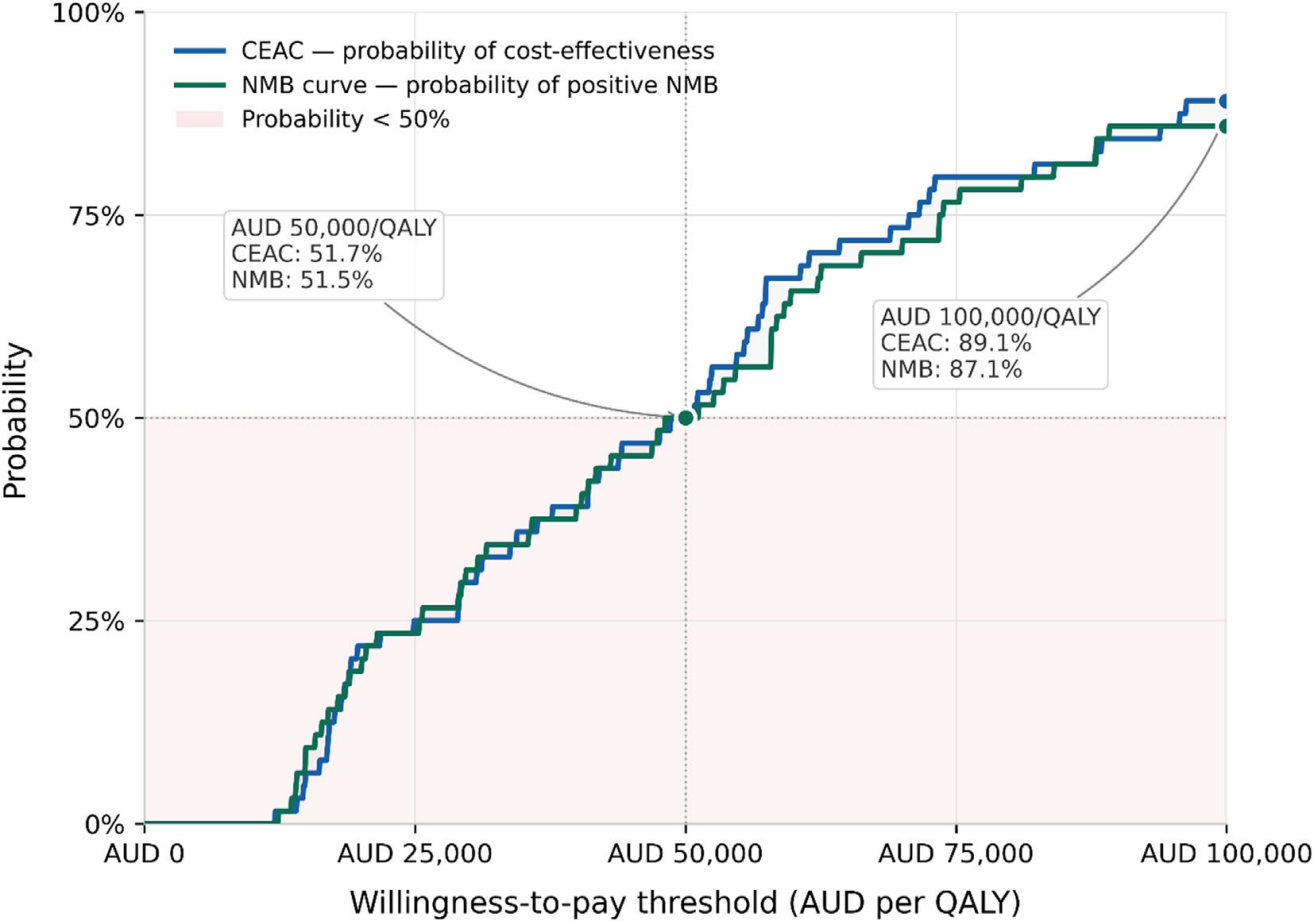
Probabilistic sensitivity analysis. Cost‐effectiveness acceptability curve (CEAC) and net monetary benefit (NMB) acceptability curve showing the probability of cost‐effectiveness and positive NMB across willingness‐to‐pay thresholds, with reference points at AUD 50 000 and AUD 100 000 per QALY.

Monte Carlo simulation (5000 iterations) showed incremental QALY gains ranging from −0.02 to 10.77 (mean 3.70) and incremental costs ranging from AUD 107 600 to AUD 164 484 (mean AUD 136 120) per patient. The cost‐effectiveness acceptability curve (CEAC) and NMB acceptability curve are presented in Figure 5. At the AUD 50 000/QALY threshold, 51.7% of simulations were cost‐effective and 51.5% demonstrated a positive NMB, with both probabilities rising to 89.1% and 87.1%, respectively, at AUD 100 000/QALY. The NMB distribution was positively skewed with a mean of AUD 49 042 (USD 31 877) but a negative median (−AUD 274), reflecting substantial uncertainty and indicating that positive economic benefits are not guaranteed across all parameter combinations.

## Discussion

This study presents an exploratory economic evaluation of NK cell therapy for paediatric solid tumors, combining primary micro‐costing of laboratory‐scale manufacturing with indicative estimates of cost‐effectiveness, headroom and NMB. The base‐case ICER of AUD 35 167 (USD 22 859) per QALY gained falls below the Australian WTP of AUD 50 000 (USD 32 500) per QALY. Economic headroom of AUD 56 386 (USD 36 651) per patient and a population NMB of ~AUD 2.8 million at 50 patients per year suggest potential economic feasibility to support further translational investigation, especially in paediatric oncology where commercial development incentives are limited and clinical need is high.^26^, ^27^, ^28^ These findings have broader implications for cellular immunotherapies, where manufacturing scalability and economic feasibility are key to clinical translation, but should be interpreted as preliminary economic signals to inform investment decisions and future modelling rather than as definitive evidence for funding decisions.

The micro‐costing analysis identifies a cost profile with direct implications for manufacturing strategy. Eighty‐eight per cent of production costs are concentrated in three steps: NK cell expansion (50.8%; AUD 29 812), mRNA transfection (25.1%; AUD 14 707) and CD3^+^ T‐cell depletion (11.7%; AUD 6864), and 85% of the cost burden comprises consumables and reagents, indicating where targeted process optimisation would have the greatest economic impact. mRNA reagents represent the largest single reagent cost at AUD 10 000 per batch, and advances in bioreactor design and reagent efficiency in the expansion step could deliver substantially greater cost reductions.^29^ The threshold analysis confirms that the economically optimal production range of 25–50 batches per year is practically achievable for a national paediatric oncology programme, and the batch consolidation scenario demonstrates that shared manufacturing runs reduce per‐batch costs by AUD 3216 and improve resilience to production failures. These manufacturing insights exemplify what early economic evaluation is designed to deliver: actionable, upstream intelligence that informs process development decisions before expensive clinical commitments are made, a translational function increasingly recognised in the early health technology assessment literature,^30^, ^31^, ^32^ but rarely applied to cellular immunotherapies.

The resource categorisation spans facility, personnel, reagents, consumables and equipment, which is aligned with established cost categories for small‐scale academic cell therapy manufacturing.^33^ It should be noted, however, that this analysis captures per‐batch production costs at laboratory scale, which are distinct from manufacturing development costs that additionally encompass facility set‐up, personnel time for process development and quality systems required to establish GMP‐compliant processes. Published analyses of cell‐based therapy manufacturing development reported facility and personnel as the dominant cost drivers, with materials comprising a minor share^33^, ^34^; in contrast to the reagent‐dominated profile identified here, where materials account for 85% of production costs. This distinction reflects both the different cost scopes and the reagent‐intensive nature of NK cell manufacturing. By comparison, academic point‐of‐care CAR‐T manufacturing has recently been costed at approximately USD 35 107 per dose, against USD 6357 per dose estimated here for NK cell manufacturing, consistent with the anticipated cost advantage of an off‐the‐shelf allogeneic platform over autologous, single‐patient manufacturing.^35^

The finding that cost‐effectiveness is driven almost entirely by QALY gains rather than manufacturing or hospitalisation costs has direct implications for future clinical trial design. A minimum QALY gain of 2.67 is required for cost‐effectiveness, equivalent to approximately 3 years of survival benefit in a population where median survival is measured in months.^36^ At the AUD 50 000/QALY threshold, both the CEAC and the NMB acceptability curve indicate a probability of only about 52%, showing that under current assumptions the intervention sits at the margin of cost‐effectiveness with substantial residual uncertainty. Notably, varying hospitalisation costs across the full plausible range (AUD 50 000–100 000) kept the ICER between AUD 28 590 and AUD 41 743 per QALY, indicating that even substantial uncertainty in this parameter does not alter the economic conclusion, provided clinical outcomes are realised.

The broader methodological challenges encountered in this analysis including limited trial data, proxy effectiveness estimates and uncertainty in health‐related quality‐of‐life inputs are well‐recognised across economic evaluations of advanced therapy medicinal products and reflect the current state of evidence for this class of therapy, not unique weaknesses of this analysis.^37^ The use of CAR‐T outcomes as a proxy is supported by several shared characteristics: Both are cellular immunotherapies requiring similar manufacturing infrastructure, both exert efficacy through immune‐mediated tumor elimination, and both represent high‐cost interventions targeting treatment‐refractory disease. However, this proxy involves substantial uncertainty, given key differences in tumor biology (haematological vs solid tumors), mechanism of action (CAR‐engineered antigen specificity vs innate NK cell recognition) and manufacturing approach (autologous vs allogeneic). In addition, sarcoma and neuroblastoma were analysed as a combined population using shared proxy effectiveness estimates, which does not account for potential differences in NK cell efficacy, tumor immunogenicity or clinical trajectory between the two tumor types. Indication‐specific analyses informed by tumor‐type‐specific data will be required as trial evidence matures. Notably, hospitalisation costs were estimated through expert opinion rather than prospective clinical micro‐costing; while the sensitivity analysis demonstrates robustness to this parameter, the central estimate remains an assumption informed by analogous allogeneic cellular therapy procedures rather than NK cell‐specific trial data. The one‐batch‐per‐patient assumption does not account for clinical heterogeneity in dosing that would emerge in practice, where disease burden, patient weight and treatment response would influence manufacturing requirements. More broadly, this laboratory‐scale model does not capture the full burden of GMP‐compliant production. Real‐world implementation would need to account for batch‐to‐batch variability and reproducibility in NK‐cell expansion, failed expansions and release failures, cryopreservation and release testing, process validation and regulatory compliance. These factors would increase the true cost per successfully treated patient, and future analyses should incorporate them as clinical development progresses. Importantly, the absence of a formal decision‐analytic model precludes assessment of long‐term cost‐effectiveness trajectories, which is especially important in paediatric populations where successful treatment could yield years of survival benefit. In the probabilistic analysis, uniform distributions were applied to cost inputs because empirical distributional data are not yet available. This is a simplification, and more informative distributions should be used as manufacturing and clinical cost data mature.

Future research should embrace the iterative early HTA approach applied here, embedding economic evaluation throughout the development pathway rather than treating it as a downstream activity. This enables manufacturing optimisation to be informed by cost data, trial design to be aligned with regulatory and reimbursement requirements, and investment decisions to be grounded in economic signals. This approach has been successfully applied to vaccines and diagnostics but remains underutilised in cellular immunotherapy, where high manufacturing costs and long development timelines make early economic intelligence particularly valuable.^22^ Manufacturing optimisation should target the three cost‐dominant steps, with a focus on reagent efficiency, process automation and scale‐up to 25–50 batches per year. Structured expert elicitation should formally quantify the most uncertain parameters such as expected survival benefit and hospitalisation costs to populate the decision‐analytic modelling.^38^

This study provides an early micro‐costing and exploratory economic evaluation of NK‐cell therapy for paediatric relapsed/refractory solid tumors. The findings identify key manufacturing cost drivers and suggest that economic feasibility will depend primarily on achieving sufficient clinical benefit, with a threshold of approximately 2.67 incremental QALYs at AUD 50 000/QALY. Given the absence of NK‐cell‐specific clinical and utility data in paediatric solid tumors, these estimates should be interpreted as preliminary decision benchmarks to guide further clinical development, manufacturing optimisation and future model‐based evaluation.

## Methods

This study composed of two related analyses. First, a cost analysis of NK cell manufacturing was conducted using primary production data to identify key cost drivers and inform process optimisation. Second, an exploratory economic evaluation was performed to generate indicative estimates of cost‐effectiveness, headroom and population net monetary benefit (NMB) for NK cell therapy in paediatric relapsed/refractory sarcoma and neuroblastoma, from an Australian health system perspective. Uncertainty and scenario analyses were conducted to test the robustness of findings across plausible parameter ranges. This study was approved by the UQ Human Research Ethics Committee (#2023/HE000027).

### Micro‐costing of NK cell manufacturing

Production costs were estimated using a structured micro‐costing approach,^39^ with primary data obtained from the Translational Research Institute (TRI) and the Frazer Institute laboratories in Brisbane, Australia. Six key manufacturing steps were identified: (1) PBMC isolation, (2) CD3^+^ T‐cell depletion, (3) mRNA transfection, (4) feeder cell expansion, (5) NK cell expansion and (6) quality control analysis. For each step, resource use was prospectively quantified and valued at 2025 market prices. Resources were categorised as instruments, equipment, consumables, reagents, personnel and facility costs. A buffy coat of 50–60 mL was assumed to yield approximately 0.5–2 × 10^8^ CD3‐depleted PBMCs, with NK cell expansion targets of 1 × 10^10^ to 2 × 10^11^ cells by Day 21 and 3 × 10^10^ to 2 × 10^12^ cells by Day 28. Each manufacturing batch was assumed to yield six doses, sufficient to treat one patient. This one‐batch‐per‐patient assumption is a key structural parameter of the cost model and was tested in scenario analysis.

### Exploratory economic evaluation

An exploratory cost‐utility analysis was conducted to generate indicative estimates of the economic value of NK cell therapy for paediatric relapsed/refractory sarcoma and neuroblastoma, compared to standard salvage chemotherapy regimens (gemcitabine/docetaxel for sarcoma; irinotecan‐based regimens for neuroblastoma) from an Australian health system perspective.^40^, ^41^ Although biologically distinct, the two tumor types were analysed as a combined population because the NK cell manufacturing process and associated costs are tumor‐agnostic and the proxy effectiveness estimates described below do not differentiate by solid tumor subtype.

#### Costs

Total cost per patient composed of two components: manufacturing costs, estimated through the micro‐costing analysis described above, and hospitalisation and administration costs. The latter was informed by local clinical expectations for delivery, monitoring and supportive care requirements for early‐phase cellular therapy administration, and by published literature on healthcare resource use and costs associated with allogeneic haematopoietic stem cell transplantation.^42^ NK cell therapy was assumed to incur lower hospitalisation costs than autologous cell therapies, given the nature of the procedure and its anticipated side effect profile, including potentially lower risk of GvHD and cytokine release syndrome, which is expected to translate to less intensive monitoring, shorter inpatient admission and reduced likelihood of intensive care unit admission.^43^ However, the toxicity profile of engineered NK‐cell products is still evolving clinically and requires prospective evaluation, and this uncertainty is reflected in the range of hospitalisation costs tested. Hospitalisation costs were estimated at AUD 75 000 per patient (range AUD 50 000–100 000), with the range reflecting uncertainty at this early stage of development. Drug acquisition and administration costs for the comparator chemotherapy regimens were below AUD 300 per cycle and were therefore excluded from the exploratory incremental cost calculation because they were small relative to the estimated NK‐cell therapy cost. This simplifying assumption does not capture the full costs of comparator administration, monitoring, toxicity management, supportive care or disease‐specific treatment pathways.

#### Effects

Given the limited number of completed clinical trials evaluating NK cell therapy in paediatric solid tumors, proxy effectiveness estimates were required. Quality‐adjusted life‐year (QALY) gains were derived from a systematic review of CAR‐T cell therapy economic evaluations in haematological malignancies,^44^ which reported a mean incremental gain of 3.80 QALYs (range 0.92–9.43) compared to standard therapy. Because NK‐cell‐specific clinical and utility data were unavailable, the proxy effectiveness estimate should be interpreted as illustrative and uncertain rather than predictive. Because cellular immunotherapy has generally shown lower efficacy in solid tumors than in haematological malignancies, this proxy most likely overestimates benefit; the magnitude of the bias is unknown and was explored through sensitivity and threshold analyses.

#### Headroom and net monetary benefit analyses

Headroom analysis was conducted to determine the maximum allowable cost per patient at which NK cell therapy could still be considered cost‐effective,^45^ calculated as follows:

Headroom=Incremental QALY gain×WTPthreshold

where the willingness‐to‐pay (WTP) threshold was set at AUD 50 000 per QALY, consistent with commonly applied benchmarks in Australian health technology assessment. This value represents the maximum justifiable cost per patient based on expected health gains alone, without accounting for potential offsetting healthcare cost savings, and therefore represents a conservative estimate of economic headroom.

The population‐level net monetary benefit (NMB) of adopting NK cell therapy across the eligible patient population was calculated as follows:

$$\mathrm{NMB}=\left(\text{Incremental QALY gain}\times \mathrm{WTP}\;\text{threshold}-\text{total cost}\right)\times V$$

where total cost includes manufacturing and hospitalisation costs from the health system perspective, and *V* is the anticipated annual patient volume. *V* was set at 50 patients per year, representing a plausible upper bound for the annual number of children in Australia with relapsed or refractory sarcoma and neuroblastoma who might be eligible for NK cell therapy.^46^ This approach enables direct estimation of the expected population‐level economic impact of adoption at an early stage of development, before formal modelled evaluation is feasible.^47^

### Uncertainty analysis

Various sensitivity analyses were conducted to characterise uncertainty in early‐stage parameter estimates. One‐way sensitivity analyses varied manufacturing cost, hospitalisation cost and incremental QALY gain individually to identify primary cost‐effectiveness drivers; two‐way analyses examined their joint effects. Threshold analyses identified minimum QALY gains required for cost‐effectiveness, maximum allowable hospitalisation costs and the relationship between annual production volume and per‐batch manufacturing costs (1–100 batches). Scenario analyses evaluated batch consolidation, whereby two patients' doses are produced within a single manufacturing run, and a 5% batch failure rate to reflect cell therapy manufacturing risk.^48^ Probabilistic sensitivity analysis was conducted using Monte Carlo simulation (5000 iterations) in R (version 4.3, R Foundation for Statistical Computing). Uniform distributions were applied to all cost inputs across plausible minimum–maximum ranges, reflecting the absence of empirical distributional data at this stage of development.^49^ QALY gains were sampled from the CAR‐T systematic review distribution,^25^, ^44^ and results are presented as acceptability curves.

## Author contributions

**Sabera Tabassum Wahid:** Methodology; investigation; formal analysis; writing – original draft. **Melissa Elliott:** Conceptualization; methodology; writing – original draft. **Yufan Wang:** Investigation; conceptualization; methodology; writing – review and editing. **Wayne Nicholls:** Conceptualization; resources. **Natacha Omer:** Conceptualization; writing – review and editing; resources. **Fernando Souza‐Fonseca‐Guimaraes:** Writing – original draft; writing – review and editing; conceptualization; funding acquisition; investigation; supervision; project administration; resources. **Haitham Tuffaha:** Resources; supervision; formal analysis; project administration; writing – review and editing; writing – original draft; funding acquisition; investigation; conceptualization.

## Conflict of interest

F.S.‐F.‐G is a Board Member of Cure Cancer Australia Foundation and a member of the Scientific Advisory Committee of ANZSA. Sanofi TSH, Microba Life Sciences and Cartherics sponsor research in the laboratory of F.S.‐F.‐G. Other authors have no commercial, proprietary or financial interest in this study.

## Data Availability

The data that support the findings of this study are available from the corresponding author upon reasonable request.
